# A systematic review and meta-analysis of clinical and functional outcomes of artificial urinary sphincter implantation in women with stress urinary incontinence

**DOI:** 10.1080/2090598X.2020.1716293

**Published:** 2020-02-04

**Authors:** Bara Barakat, Knut Franke, Sameh Hijazi, Samer Schakaki, Ulrich Gauger, Viktoria Hasselhof, Thomas-Alexander Vögeli

**Affiliations:** a Department of Urology and Pediatric Urology, Hospital Viersen, Viersen, Germany; b Department of Urology, Hospital Ibbenbüren, Ibbenbüren, Germany; c Department of Urology, Hospital Osnabrück, Osnabrück, Germany; d Private Medical Statistics, Berlin, Germany; e St. Elizabeth Boardman Family Medicine, Boardman, OH, USA; f Department of Urology and Pediatric Urology, University Hospital RWTH Aachen, Aachen, Germany

**Keywords:** Artificial urinary sphincter, urinary incontinence, stress urinary incontinence

## Abstract

**Objective:**

To evaluate the complications and results of artificial urinary sphincter (AUS) implantation in women with stress urinary incontinence (SUI).

**Methods:**

A selective database search using keywords (1990–2019) was conducted to validate the effectiveness of the AUS in women. Preferred Reporting Items for Systematic Reviews and Meta-Analyses (PRISMA) guidelines were utilised. The meta-analysis included 964 women (15 studies) with persistent SUI. The Newcastle-Ottawa score was used to determine the quality of the evidence in each study. The success rate and complications associated with the AUS were analysed.

**Results:**

Meta-analysis of the published studies showed that complete continence was achieved at a mean rate of 79.6% (95% confidence interval [CI] 72.2–86.6%) and a significant improvement was achieved in 15% (95% CI 10–25%). The mean (range) follow-up was 22 (6–204) months. The mean number of patients per study was 68. The mean (range) explantation rate was 13 (0–44)%. Vaginal erosion occurred in a mean (range) of 9 (0–27)% and mechanical complications in 13 (0–47)%. Infections accounted for 7% of the complications. The total mean (range) revision rate of the implanted AUS was 15.42 (0–44)%. The mean (range) size of the cuff used was 6.7 (5–10) cm.

**Conclusion:**

Our present analysis showed that implantation of an AUS in women with severe UI is an effective treatment option after failure of first-line therapy. However, the currently available study population is too small to draw firm conclusions.

**Abbreviations:**

AMS: American Medical Systems; AUS: artificial urinary sphincter; EAU: European Association of Urology; LE: Level of Evidence; PRISMA: Preferred Reporting Items for Systematic Reviews and Meta-Analyses; QoL: quality of life; SHELTER: Services and Health for Elderly in Long TERm care (study); SUI: (stress) urinary incontinence

## Introduction

Stress urinary incontinence (SUI) is an increasing problem for women and it affects both their quality of life (QoL) and that of their loved ones. It is a problem that results in economic and financial burdens. According to recent studies, the prevalence of SUI in women aged >40 years lies between 20% and 36% [1]. In an European multicentre study (the Services and Health for Elderly in Long TERm care [SHELTER] study) of 4156 nursing home patients (472 from Germany), 73.5% where found to have UI [2]. The incidence of UI is expected to increase further with an ageing population [3]. Medical treatment of moderate-to-severe SUI is inferior to other treatment options due to its limited efficacy. Surgical treatment of female SUI with the use of alloplastic bands seems to have short-term effectiveness. However, the United States Food and Drug Administration (FDA) has published warnings about the use of alloplastic implants for the surgical treatment of female UI on several occasions, due to >1000 reported severe side-effects from its use [4].

Consequently, physicians treating female SUI are extremely interested in obtaining reliable data on therapy options and their effectiveness. Foley was the first to describe an AUS in 1947 [5,6]. The AUS consisted of an air-inflatable, periurethral cuff that was attached to a removable pump, but this system proved to be unsuitable in practice. Multiple other systems were developed in the following decades; however, most were not clinically implemented. The first AUS was developed by Bradley and Scott (Scott- AS721) in 1972 and was produced by American Medical Systems (AMS) Inc. (Minnetonka, MN, USA) [7]. Their AUS system consisted of a urethral cuff, two pumps to fill and empty the cuff, a reservoir, and four valves [7]. The Model AS792 (792: bladder neck placement) was developed in 1979. It consisted of three components, including the current version of the pressure-regulating balloon. This version did not contain deactivating buttons, which lead to a high rate of urethral erosions, particularly in the early postoperative period. Over time, further development of the AUS lead to a safe and reliable treatment option for SUI. While the AUS became the ‘gold standard’ for the treatment of male moderate-to-severe SUI [8], its use in women remained limited.

The international urological societies, including the AUA, the European Association of Urology (EAU) and the ICS recommend the implantation of an AUS as a second-line treatment option for SUI due to a lack of evidence-based long-term data [9,10]. The National Institute for Health and Care Excellence (NICE), also recommend implantation of an AUS as second-line treatment after failed prior surgical treatment in their 2015 guidelines [11]. The above-mentioned guidelines and societies all criticise the lack of randomised control trials with regard to AUS implantation. Furthermore, the exact indication and optimal timing of AUS implantation in women are unknown. Consequently, the decision of when and in whom to implant an AUS is influenced by the prior experience of the treating surgeon.

The implantation of an AUS for the treatment of male SUI has good long-term data on the continence rate (79%) and high patient satisfaction in one meta-analysis [12,13]. However, this long-term data does not exist for the treatment of female SUI. Data suggest that ~25% of women required a revision operation after good initial results and that the rate of revision increased with time [14].

The focus of the present study, was to determine the complication rate, continence rate, and long-term results of AUS therapy for women with SUI.

## Patients and methods

### Meta-analysis criteria

A selective literature review of PubMed, National Library of Medicine, the Medical Literature Analysis and Retrieval System Online (MEDLINE), Cochrane Central Register of Controlled Trials (CENTRAL), Google scholar and Clinical Trials was conducted by searching for studies addressing SUI by implantation of an AUS. Studies published from 1990 to 10 October 2019, containing cohorts of women who received an AUS for the treatment of SUI were included. The following keywords were used to search for appropriate studies: ‘urinary incontinence’, ‘female urethral sphincter’, ‘artificial sphincter’, ‘female artificial sphincter’, ‘AMS sphincter 800ʹ.

The protocol with inclusion criteria of this work was registered by International Prospective Register of Systematic Reviews (PROSPERO: CRD42019118386). The included studies all addressed the effectiveness of the AUS, continence rate, and long-term results. The search was limited to published articles, systematic reviews, and original works. Reference lists of the included articles were also reviewed for relevant articles. The articles were selected by review of the abstract and subsequently reviewed in detail. Included articles were selected by consensus of all authors. Two independent researchers reviewed the articles before the final consensus decision was made to include them in this meta-analysis.

### Data extraction and quality evaluation

The following information was extracted from the studies that met the inclusion criteria: name of the first author, year of publication, study design, patients’ demographic data, type of intervention, follow-up data, perioperative data, surgical results, and complications (vaginal/urethral erosions, infection, bladder injury, surgical revision rate, and explantation rate). These details were then examined to determine the potential of performing subgroup analyses. The systematic review and meta-analysis were conducted in accordance with the Preferred Reporting Items for Systematic Reviews and Meta-Analyses (PRISMA) guidelines [15]. The 9-point Newcastle-Ottawa Cohort Study Scale was used to assess the quality of the included studies [16]. The articles were graded according to selection (4 points), comparability (2 points), and results (3 points). Published studies that received >6 points were considered to be of high quality. Two authors independently reviewed the articles for final approval before the final consensus decision was made to include them in this meta-analysis.

### Statistical analysis and data synthesis

Relevant demographic and functional endpoints were extracted from the included studies, including total patients, study design, mean patient age, surgical outcomes, and complications.

The primary outcome of this meta-analysis was the percentage of continent and partially continent patients after surgery. Furthermore, we recorded prior operations, accompanying operations, follow-ups, revision rates, and complication types (bladder injury, erosion, infection). Outcomes related to SUI were summarised based on preoperative UI status, as we considered this to be the most important factor in the development of postoperative disease. If a study did not include a mean and standard deviation (SD), we used the median and sample size to estimate the mean and variance. All analyses were performed using the Comprehensive Meta-Analysis statistical analysis software, version 2.0 (Biostat, Englewood, NJ, USA).

## Results

### Study selection, study characteristics and outcomes

**Figure 1.**
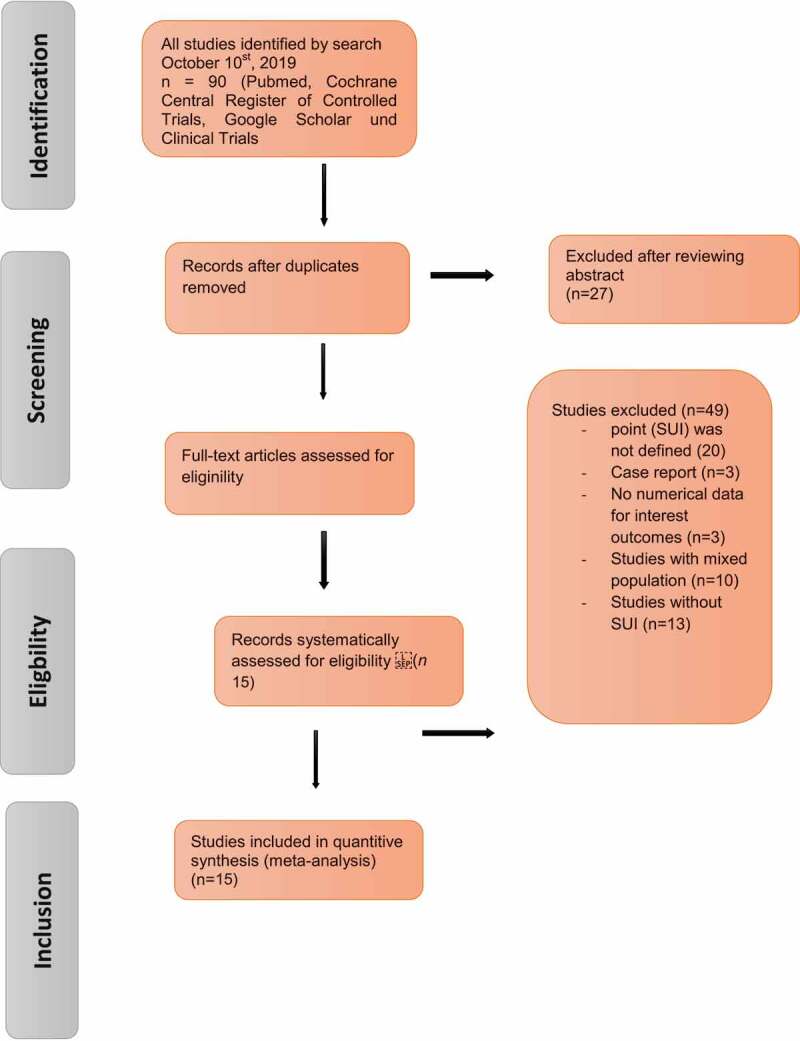
Study selection flow chart, systematic PRISMA search strategy.

The search strategy is described in the PRISMA flowchart and the results of the literature review for the systematic review are illustrated in Figure 1. After de-duplication, 90 articles were screened for further analysis and 15 studies (964 women) identified (Table 1 [14,17–30]), which included cohorts of patients undergoing AUS implantation. In particular, the studies that fit the Population, Intervention, Control, Outcome (PICO) quality assessment criteria were included in this analysis. Of the 90 relevant articles, 15 (964 women) met the criteria for inclusion in this analysis (Figure 1, Table 1).

**Table 1.** Summary of basic characteristics and perioperative outcomes of selected studies for meta-analysis.

Reference	Operation access	Patients, *n*	Age, years, mean	Previous anti-UI surgery, % (*n/N*)	Previous pelvic surgery, % (*n/N*)	MUCP, cm	Length of hospital stay, days, mean	Erosion, % (*n/N*)	Urethral, bladder injury, % (*n/N*)	Infection, % (*n/N*)	Cuff size, cm, mean (range)
Tricard et al. 2019 [17]	Open	63	58	18 (11/63)	82 (52/63)	27	9	8 (5/63)	3 (2/63)	NR	NR
Gondran-Tellier et al. 2019 [18]	Robotic	8	67	NR	NR	24	5	(0/8)	(0/8)	(0/8)	NR
Peyronnet et al. 2018 [19]	Robotic	49	70.5	85.7 (42/49)	NR	24	4	6 (3/49)	10 (5/49)	NR	7 (5–9)
Ferreira et al. 2017 [20]	LAP	52	69	79 (39/52)	98 (51/52)	16	2	8 (4/52)	(0/49)	2 (1/52)	7 (6–7)
Phé et al. 2014 [14]	Open	34	56	97 (33/34)	97 (33/34)	19	11	(0/34)	15 (5/34)	NR	7 (5–10)
Biardeau et al. 2015 [21]	Robotic	11	66	(11/11)	(5/11)	28	5	(2/11)	(2/11)	(0/11)	6 (5–7)
Fournier et al. 2014 [22]	Robotic	6	65	(5/6)	NR	16	6	(0/6)	NR	(0/6)	8 (7–9)
Costa et al. 2013 [23]	Open	344	57	69 (261/344)	43 (163/344)	NR	NR	25 (12/344)	6 (17/344)	18 (61/344)	NR
Trolliet et al. 2013 [24]	LAP	26	64	100 (26/26)	81(21/26)	26	6	27 (7/26)	19 (5/26)	0 (0/26)	6 (5–8)
Vayleux et al. 2011 [25]	Open	215	63	89 (184/215)	93 (200/215)	24	NR	5 (11/215)	11 (24/215)	1 (3/215)	7 (6–7)
Chung et al. 2010 [26]	Open	47	51	74 (35/47)	70 (33/47)	NR	NR	17 (8/47)	NR	4 (2/47)	NR
Mandron et al. 2010 [27]	LAP	25	67	84 (21/25)	36 (9/25)	16	4	8 (2/25)	(0/25)	(0/25)	6 (5–8)
Rouprêt et al. 2009 [28]	LAP	12	57	(11/12)	(5/12)	24	7	(2/12)	(2/12)	NR	7 (6–7)
Ngninkeu et al. 2005 [29]	LAP	4	68	(2/4)	(1/4)	24	8	(0/4)	(0/4)	NR	NR
Thomas et al. 2002 [30]	Open	68	51	81 (55/68)	73 (50/68)	NR	NR	NR	46 (31/68)	46 (31/68)	NR

MUCP, maximum urethral closure pressures; LAP, laparoscopic; NR, not reported.

The evaluated study designs were retrospective and prospective analyses of cohorts of women who underwent implantation of an AUS. The number of operating surgeons was not quantified in the included studies. There were no randomised studies that met the ICS recommendations for clinical research on implantable surgical devices [16]. The primary endpoint for the included studies at the last follow-up was continence categorised as: complete continence (no leaking, no pads used), social continence (1–2 pads/day), improved incontinence (>50% decrease in number of pads used), or failure (<50% improvement, persistent or increased leaking). The secondary endpoints were: any complications, explantations, and revision-free time. The evaluation of patients often did not include validated instruments to quantify continence-related QoL. The inherent weaknesses of this type of study include loss of follow-up bias and recall bias. Therefore, all studies were rated according to the Newcastle-Ottawa Scale grading system with 3–7 stars (out of a maximum of 9 stars) [31].

### Excluded studies

Initially, our search yielded 90 publications. After removal of the duplicates, 27 articles remained and were screened using their title and abstract; leaving 63 articles selected for full-text review. Another 25 studies were excluded from the meta-analysis because an endpoint (SUI) was not defined and mentioned in the context (*n* = 20) or they were a case report (*n* = 5). Three studies were excluded because the results were not quantitatively reported or UI was not defined. Another 10 studies were excluded due to a mixed and paediatric population; and 14 were excluded as they investigated neurogenic UI. The flow of studies through the selection process is presented in Figure 1.

### Quality of the evidence

The rating of very low-quality evidence per outcome across trials was based on the judgement of serious limitations (risk of bias), serious imprecision and likely publication bias in all the outcomes across trials. All 15 studies identified in this review were rated at high risk of bias. The hierarchy’s rank of included studies according to the probability of bias was Level of Evidence (LE) IV.

### Patient characteristics and clinical features

The patients’ clinical and surgical histories are presented in Table 1. All the included studies were of women with moderate and severe SUI. The median (range) age of the patients included in these studies was 64 (56–70.5) years. The median (range) follow-up time was 22 (6–204) months. The mean number of patients per study was 68. Six of the included studies reported on an open approach, nine studies reported on a minimally invasive approach (laparoscopic *n* = 5, robot-assisted surgery *n* = 4). Continence surgery was performed in conjunction with prolapse surgery in six studies. In all, 65% of all the patients had a history of previous pelvic surgery and 78% had previously undergone unsuccessful anti-UI procedures. The mean (range) maximal urethral closure pressure was 27 (9–50) cm H_2_O.

### Perioperative outcomes

The mean (range) length of hospital stay was 6 (2–11) days after surgery. The perioperative documented compilations were: infection; urethral, vaginal and bladder neck injuries. The mean (range) infection rate was 7 (0–46%). Four studies did not report an infection rate. Costa et al. [23] reported a device infection risk of 4.8%. Apart from infections, urethral erosion of the AUS had a serious impact on the success of the operation. The mean (range) AMS erosion rate was 9 (0–27)% and the mean bladder injury rate was 11 (0–46)%. The mean (range) explantation rate was 13 (0–44)%. The mean (range) overall revision rate of the implanted AUS was 15.42 (0–44)%. The mean size of the cuffs used was 6.7 (5–10) cm.

### Short- and long-term functional outcomes and QoL

All included studies provided numerical data on daily postoperative pad use. However, interpretation and direct comparison were difficult due to variability in surgical techniques and selection of patients with different UI severity. The evaluation of the patients included pad tests and did not use validated instruments to quantify continence-related QoL. The median postoperative cure rate (continence rate: no pads) was 77 (61–94%) and the postoperative social continence rate (continence rate: 1–2 pads) was 22 (10–74%) (Table 2 [14,17–30]).

**Table 2.** Short/long-term clinical outcomes, rates of surgical revision of selected studies.

Reference	Follow-up, months, mean	Fully continent (no pads), % (*n/N*)	Social continence (1–2 pads), % (*n/N*)	Improved continence, % (*n/N*)	Mechanical complications, % (*n/N*)	Revision rate, % (*n/N*)	Explantation rate, % (*n/N*)	Device survival rate, years/%
Tricard et al. 2019 [17]	180	81 (51/63)	NR	9 (6/63)	5 (3/63)	5 (3/63)	8 (5/63)	20/69
Gondran-Tellier et al. 2019 [18]	12	(5/8)	(3/8)	(8/8)	(0/8)	(0/8)	(0/8)	NR
Peyronnet et al. 2018 [19]	18	81 (40/49)	12 (6/49)	12 (6/49)	2 (1/49)	6 (3/49)	2 (1/49)	NR
Ferreira et al. 2017 [20]	37	73 (38/52)	16 (8/52)	94 (46/49)	12 (6/52)	21 (11/52)	21 (11/52)	2/87 5/78
Phé et al. 2014 [14]	204	61 (21/34)	14 (5/34)	NR	47 (16/34)	34 (12/34)	24 (8/34)	10/80 15/80 20/74
Biardeau et al. 2015 [21]	19	(7/8)	(1/8)	(6/8)	NR	(3/8)	NR	NR
Fournier et al. 2014 [22]	14	(5/6)	(1/6)	(6/6)	(0/6)	(0/6)	NR	NR
Costa et al. 2013 [23]	108	94 (322/344)	10 (33/344)	NR	15 (51/344)	6 (22/344)	NR	3/90 5/89 10/69
Trolliet et al. 2013 [24]	20	61 (16/26)	19 (5/26)	19 (5/26)	8 (2/26)	15 (4/26)	NR	NR
Vayleux et al. 2011 [25]	72	66 (143/215)	74 (159/215)	NR	15 (33/215)	15 (33/215)	7 (15/215)	NR
Chung et al. 2010 [26]	162	85 (40/47)	NR	NR	38 (18/47) after 20 years	34 (16/47) after 20 years	17 (8/47)	8/80
Mandron et al. 2010 [27]	26	83 (19/23)	17 (4/23)	NR	(0/25)	(0/25)	8 (2/25)	NR
Rouprêt et al. 2009 [28]	12	(10/12)	(2/12)	(12/12)	NR	(0/12)	NR	NR
Ngninkeu et al. 2005 [29]	6	(3/4)	(1/4)	(4/4)	NR	(1/4)	(0/4)	NR
Thomas et al. 2002 [30]	84	81 (55/68)	NR	NR	18 (12/68)	18 (12/68)	44 (30/68)	NR

NR, not reported.

**Figure 2.**
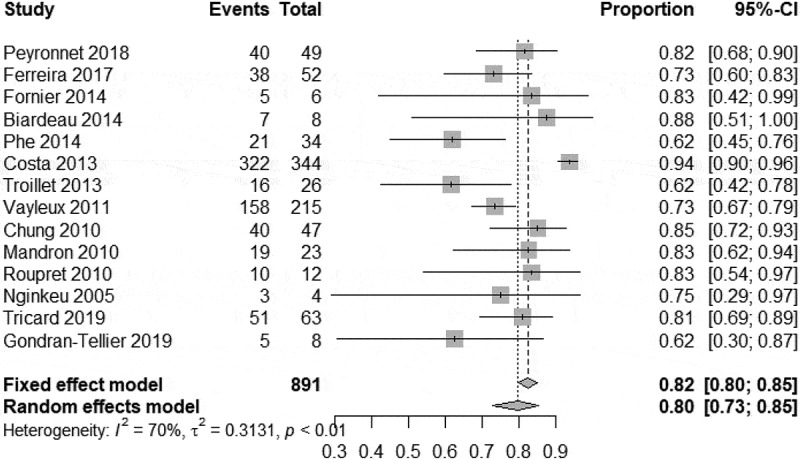
Meta-analysis; heterogeneity of the continence rate 79.6% (95% CI 72.2–86.6%).

**Figure 3.**
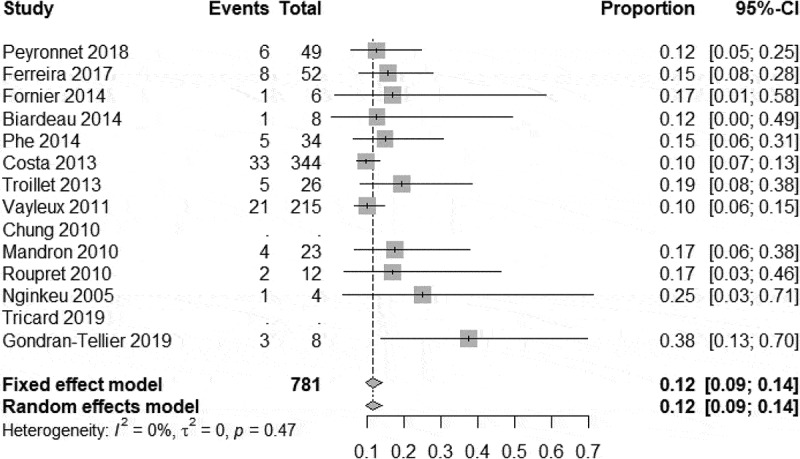
Meta-analysis; heterogeneity of the social continence rate 11.4% (95% CI 9.3–13.9%).

In our meta-analysis, we found a high degree of heterogeneity of the continence rate 79.6% (95% CI 72.2–86.6%) (Figure 2). Therefore, the total continence rate after implantation of an AUS, despite multiple previous operations, was 80% in our analysis. In all studies analysed, there was no heterogeneity between studies in terms of social continence. The meta-analysis on social continence following AUS implantation was 11.4% (95% CI 9.3–13.9%) (Figure 3). Three of these studies with long-term outcomes reported a continence rate of 80–89% after 5 years[14, 23].

### Short- and long-term outcomes of surgical revision

The major AUS complications noted during long-term follow-up were prosthetic infections, mechanical problems, urethral erosion resulting in recurrent UI, and subsequent revision or explantation of devices. In our meta-analysis, we found a significant revision rate of 15.42 (0–44)%. The survival rate decreased with longer follow-up and the revision rate increased over time. Ferreira et al. [20] and Costa et al. [23] reported 5-year functional rates of the primary implanted AUS to be 78% and 89%. Costa et al. [23] and Phé et al. [14] reported 10-year functional rates to be 69.2% and 80%. In a single-institution study of female AUS with a long follow-up of up to 20 years, device survival rates at 10, 15, and 20 years were 79%, 65%, and 38%, respectively [14]. These numbers are meaningful and show a long period of improvement in QoL. Overall, the complication rates were manageable, but the need for revision surgery increased over time. All revisions were due to the mechanical failure of the pump, the balloon, the cuff or the connections. However, it should be noted that the variability in the number of patients per study was very heterogeneous and the published studies with longer follow-up were very few. The authors report that in most cases, mechanical failure was the reason for a revision. The mean (range) explantation rate in our analysis was 13 (0–44)%. The explantation rate was not reported in five studies.

### Device survival outcomes and mechanical failures

Overall, our analysis found an incidence rate of mechanical failure of up to 13%. Three studies did not report on mechanical failure of the device. Ferreira et al. [20] reported an AUS survival rate of 87.1% and 78.9% at 2 and 5 years of follow-up, respectively. The mean interval between implantation and the first device defect or first complication was 42.1 months.

### Subgroup analysis and investigation of heterogeneity

In a subgroup analysis using the fixed-effects model, we found no significant heterogeneity between surgical procedures (open, minimally invasive approach) for functional outcomes: continence rate (random effects variance 1.56, 95% CI 0.19–0.168; *P* > 0.5). We found significant heterogeneity between surgical procedures (open, minimally invasive approach) for device outcomes: explantation rates (random effects variance 0.048, 95% CI 0.019–0.0168; *P* < 0.012).

## Discussion

While the AUS has become the ‘gold standard’ for the treatment of male moderate-to-severe SUI, the implantation of the same sphincter has been limited in women [8]. There is a broad therapeutic spectrum for the treatment of female UI, which allows for individualisation of the therapy. Generally, other surgical methods are used first on women because they are less invasive and have a lower risk of complications. Despite convincing functional results, the implantation of a prosthesis in women is usually used as a second-line therapy only after failed previous surgical treatment [11]. Several new studies have been published,especially in recent years with different approaches. However, most of the literature consists of retrospective studies of heterogeneous groups and different definitions of improvement or success, making it difficult to directly compare studies. To date, there is no evidence for the effectiveness of the AUS in women with SUI. The pooled results of the AUS were calculated, including the risk difference for AUS revision surgery. The main reasons for a revision were quantified as much as possible. Chartier-Kastler et al. [32] reported in their work that the complication rate of the published studies after 1999 were more favourable. This was because technical changes to the surgical procedure have since been made. The rate of non-mechanical complications was reduced from 16.5% to 8.8%.

In our present meta-analysis, we found that 83% of women had received prior pelvic and anti-UI surgery. Having had more than one previous surgery for UI has been associated with an increased risk of explantation of the device due to non-mechanical complications [32]. The results of AUS implantation for the treatment of UI are good, despite the patients having severe forms and having often undergone several previous operations. In general, the success rates varied between 61% and 86%. The total continence rate after implantation of an AMS sphincter, despite multiple previous surgeries, was 80% in our present analysis. A small portion of the patients (15.42%) required a revision surgery, with the revision rate increasing over time. Although the functional lifetime of the device has been well documented, a significant percentage of patients had to have a revision for various reasons including: erosion, infection, and mechanical failure. In one single-institution study with a long follow-up of up to 20 years, device survival rates at 10, 15, and 20 years were 79%, 65%, and 40%, respectively [14]. Phé et al. [14], Costa et al. [23], Ferreira et al. [20] found that the cumulative risk of device explantation increased over time. Most studies had different follow-up time-frames and often <5 years of follow-up. The mean (range) percentage of infection in all studies was 7% (0–46%). Vayleux et al. [25] reported a 15.3% incidence of device failure in one study of female patients. They identified several risk factors for device failure, which subsequently led to the explantation of the AMS AUS. According to the authors, prior irradiation of the pelvis, age >70 years, and previous surgeries, were high risk factors for re-operations. In the case of a minimally invasive approach (laparoscopic *n* = 5, robot-assisted surgery *n* = 4) the continence rate ranged from 61% to 86%, but the follow-up was shorter and fewer patients were included compared to the open approach. In the future we need to compare the effectiveness and safety of the different surgical techniques.

Finally, most authors emphasised that AUS implantation is more complicated than a sling procedure, so the surgeon’s experience plays a crucial role in the success rate. The principal limitation of the present review is the absence of the use of a validated UI questionnaire and validated instruments to quantify continence-related QoL. All 15 studies identified in the present review were rated at high risk of bias and of poor quality according to the Newcastle-Ottawa Scale grading system. The hierarchies rank of included studies according to the probability of bias was LE IV. Most of the studies included in the present meta-analysis were retrospective cohorts and therefore the results must be interpreted with caution. Although a considerable number of studies were included, some were susceptible to follow-up with revision and explantation. However, due to the long follow-up period, we believe that the results of the present study should be considered. The present study confirmed the effectiveness and excellent mechanical survival of AUS in women. For this reason, AUS implantation should be considered as a good alternative therapy for the treatment of UI.

## Conclusion

In summary, the AUS provided satisfactory long-term functional outcomes with manageable complications in women with UI, most of which were caused by significant intrinsic sphincter dysfunction following the failure of previous surgical methods. As expected, the results worsened after 10 years. However, it is worth noting that >60% of women remained continent, even though the device had a lifespan of less than a decade. Despite the good results obtained with the AUS, there is a relatively high need for revision surgery. The best-known risk factors for explantation are: ageing, previous procedures, perioperative complications, and pelvic radiation [30, 32–35]. In conclusion, further prospective studies are needed comparing open, laparoscopic and robot-assisted surgery, with the evaluation of surgery times, safety, and morbidity and mortality

## Supporting information

PRISMA 2009 checklist.
